# Construction and characterization of bioluminescent *Salmonella* Reading outbreak and non-outbreak strains

**DOI:** 10.1128/spectrum.01263-24

**Published:** 2024-12-27

**Authors:** Abubakar Shitu Isah, Reshma Ramachandran, Anuraj Theradiyil Sukumaran, Aaron S. Kiess, Claudia D. Castañeda, Timothy Boltz, Kenneth S. Macklin, Hossam Abdelhamed, Li Zhang

**Affiliations:** 1 Department of Poultry Science, Mississippi State University, Mississippi State, Mississippi, USA; 2 IDEXX Laboratories, One IDEXX Drive, Westbrook, Maine, USA; 3 Freshpet, Bethlehem, Pennsylvania, USA; 4 Prestage Department of Poultry Science, North Carolina State University, Raleigh, North Carolina, USA; 5 Engrain LLC, Manhattan, Kansas, USA; 6 Department of Comparative Biomedical Sciences, College Veterinary Medicine, Mississippi State University, Mississippi State, Mississippi, USA; University of Minnesota Twin Cities, St. Paul, Minnesota, USA

**Keywords:** *Salmonella *Reading, foodborne pathogen, outbreaks, transformation, bioluminescence imaging

## Abstract

*Salmonella* enterica serotype Reading has recently been identified as a significant foodborne pathogen from contaminated poultry products. There is a critical need for close monitoring of this newly emerged pathogen. This study developed bioluminescent strains of *S.* Reading for real-time pathogen tracking using bioluminescence imaging. Two strains of *S*. Reading were used: an outbreak strain and a non-outbreak strain. The chloramphenicol acetyltransferase gene was cloned into the plasmid pBS-*slp*GFP*luxABCDE*, which carries *luxABCDE* operon and an ampicillin resistance gene. The newly constructed plasmid was then transformed into the outbreak and non-outbreak strains of *S*. Reading by electroporation. The resulting colonies were confirmed by visualizing bioluminescence using an *in vivo* imaging system and by testing their resistance to chloramphenicol. These strains demonstrated a high bioluminescence level (10^8^–10^9^ Photons/s/cm^2^/sr) and were tested for growth and plasmid stability by daily subculturing in Luria–Bertani medium with and without antibiotics. The plasmid remained stable for 8 days under non-selective conditions, and growth rates were comparable to non-bioluminescent parent strains in antibiotic-free conditions. However, growth was notably different in the presence of chloramphenicol, indicating successful plasmid retention and function. This study successfully created stable bioluminescent *S*. Reading strains, marking a significant step forward in monitoring and potentially reducing the spread of this emergent foodborne pathogen in the poultry industry.

**IMPORTANCE:**

*Salmonella enterica* serotype Reading has recently become a significant foodborne pathogen linked to poultry products. To enhance pathogen monitoring, this study developed bioluminescent strains of *S.* Reading by inserting the chloramphenicol acetyltransferase gene into a plasmid containing a bioluminescence gene cluster. These modified strains were transformed into outbreak and non-outbreak bacterial strains via electroporation. The bioluminescent strains demonstrated stable plasmid retention and high bioluminescence levels. They also showed growth comparable to their parent strains, even in the absence of antibiotics. These bioluminescent strains could potentially facilitate real-time monitoring and control of *S.* Reading in poultry industries.

## INTRODUCTION

The genus *Salmonella* belongs to the family Enterobacteriaceae consisting of two main species, six subspecies, and over 2,600 serotypes (1). The *Salmonella enterica* serovars are commonly connected to infections in people and other warm-blooded animals, causing typhoidal and non-typhoidal Salmonellosis (2). Salmonellosis rarely causes clinical disease in poultry, and most infected birds remain asymptomatic carriers. However, Salmonellosis is a major cause of foodborne illness in humans both in the United States and globally, with poultry as the major source of infection (2–5). The most often isolated serotypes from poultry and poultry products in the United States are Kentucky, Enteritidis, Infantis, Senftenberg, Schwarzengrund, Heidelberg, Hadar, Montevideo, Mbandaka, Thompson, and Typhimurium (6).

*S. enterica* subsp. *enterica* serotype Reading (*S*. Reading) recently emerged as a major foodborne pathogen causing two separate, large, multistate outbreaks in North America (7). The United States and Canada have recently observed an increase in cases with this rarely isolated serotype. In the United States alone, this serotype resulted in 358 illnesses across 42 states, leading to 133 hospitalizations resulting in the recall of poultry products. The outbreak investigation by the United States and Canada identified turkeys and chicken products as sources of the pathogen (8–10).

Due to the recent increase in outbreaks caused by *S*. Reading, there is a need to find effective methods for monitoring this emergent serotype in live poultry and poultry products. Additionally, understanding the effectiveness of the sanitation process in poultry processing plants is crucial. For this purpose, we chose to create bioluminescent *S*. Reading strains. Bioluminescence resulting from the expression of the *lux*CDABE operon is a phenomenon where bacteria emit light that is detectable by bioluminescent imaging (BLI). Real-time tracking and monitoring of bacterial presence can be accomplished using the BLI (11–13). In addition, the study has shown that *Salmonella* strains engineered to express bacterial luciferase did not exhibit any observable difference in their growth dynamics (14, 15).

In this study, we aimed to develop and characterize two bioluminescent strains of *S*. Reading. These included the recent outbreak strain (SRO) and a non-outbreak reference strain (SRN), as identified by Ref. (7), which can be utilized for food safety monitoring of the emerging serotype in live poultry and poultry products (7).

## MATERIALS AND METHODS

### Bacterial strains, growth conditions, and plasmids

Table 1 lists the bacterial strains and plasmids used in this research. All the *S*. Reading strains were recovered from commercial turkey barns. The *S*. Reading non-outbreak strain (SRN)(RS326; NCBI: SAMN13874562) rarely causes illness in humans, whereas the *S*. Reading outbreak strain (SRO) (RS330; NCBI: SAMN13874566) is the emergent strain from the recent human outbreaks in North America. Both strains were donated by Dr. Timothy Johnson (7). Throughout the study, *S*. Reading strains were cultured at 37°C for 24 h in either xylose lysine tergitol 4 (XLT4) agar (BD Difco) or Luria–Bertani (LB) agar and broth (Difco). The *Escherichia coli* TOP10 strain was grown at 37°C for 24 h in LB agar and broth (Difco). When required, antibiotics were added to the culture medium at concentrations of 200 µg/mL for ampicillin and 10 µg/mL for chloramphenicol.

**TABLE 1:** Bacterial strains and plasmids used in this study^*a*^

Strains and plasmids	Description	Sources/References
Bacterial strains		
*S*. Reading 326 (reference non-outbreak)	Historical strain	(7, 16, NCBI: SAMN13874566)
*S*. Reading 330 (outbreak)	Emergent strain	(7, 16, NCBI: SAMN13874562)
*Escherichia coli* TOP10	Competent cell	Thermo Fisher
Plasmids		
pBS-*slp*GFP*luxABCDE*	Vector: *lux*, *amp*	(13)
pMJH46	Donor: *cat*	(17, Addgene plasmid:67272)
pBS-*slp*GFP*lux-cat*	Construct: pBS-*slp*GFP*luxABCDE*, *cat*	This study

^*a*^ *cat*: chloramphenicol acetyltransferase (chloramphenicol resistance gene); *amp*: ampicillin resistance gene; *lux:* bacterial luxCDABE operon.

### Construction of pBS-*slp*GFP*lux-cat*

While the SRO strain is resistant to ampicillin and sensitive to chloramphenicol, the SRN strain is sensitive to both antibiotics. Consequently, the bioluminescence expression vector plasmid pBS-*slp*GFP*luxABCDE* (13), which only carries the ampicillin resistance gene (*amp*), could not be used to transform both strains. Instead, chloramphenicol resistance gene (*cat*) was cloned into the plasmid, retaining the existing *amp*. The cat gene was amplified from a donor plasmid pMJH46 (16, Addgene plasmid: 67272) using two primers. Primer R: AAA*GAGCTC*TCGAGATTTTCAGGAGCTAAGG and Primer F: AAA*ACTAGT*AGGGCACCAATAACTGCCTTA each carried SpeI and SacI restriction enzyme sites (italicized bases) at their 5′ ends, respectively. The PCR-ampliﬁed fragment (717 bp) was then ligated into recipient plasmid pBS-*slp*GFP*luxABCDE*, which had been digested with the same enzymes, yielding the new plasmid named pBS-*slp*GFP*lux-cat*. The cloning step was followed by the transformation of the *E. coli* TOP10 strain with the new plasmid. Bioluminescence expression and chloramphenicol resistance in *E. coli* were confirmed by growth in LB agar containing chloramphenicol and bioluminescence detection using *in vivo* imaging. The plasmid was then extracted from the *E. coli* using the QIAprep Spin Miniprep Kit (Qiagen, Hilden, Germany) in accordance with the instructions.

### Making *S*. Reading strains electrocompetent

*S*. Reading strains were made electrocompetent and potent for electroporation by streaking strains in LB agar plates and incubating at 37°C for 24 h. A single colony was then picked and inoculated into 5 mL LB broth and incubated at 37°C for 24 h with 200 rpm shaking. One hundred-fold (1:100) dilution of the overnight culture was then prepared and grown at 37°C with shaking at 200 rpm until the large culture reached OD _600_ ~0.75. Cells were chilled in an ice water bath for 15 min and pelleted by centrifuging at 4,000×*g* for 10 min at 4°C. The pellets were resuspended and washed in 10% cold glycerol solution four times to make the *S*. Reading electrocompetent. For each washing, 10% of fresh cold glycerol was used to resuspend the pellet, and then centrifuged at 4,000×*g* for 10 min at 4°C. The final pellet was then reconstituted in 1 mL of 10% cold glycerol and split into 100 µL volumes in 1.5 mL tubes and stored at −80°C until used for transformation.

### Transformation of electrocompetent *Salmonella* Reading strains

The newly constructed plasmid *pBS-slp*GFP*lux-cat* was used to transform the electrocompetent *S*. Reading strains by electroporation by mixing the 5 µL of plasmid with 100 µL of electrocompetent cells in a prechilled cuvette, and then exposing the mixture to 2,500 V in 5 ms using the BTX Harvard Apparatus ECM 399 Electroporation System. The electroporated strains were then recovered in SOC media and incubated at 37°C for 1 h before being spread-plated on LB agar supplemented with chloramphenicol. After 24 h of incubation, colonies were viewed for the presence of bioluminescence using IVIS Lumina XRMS *in vivo* Imaging System Series III (PerkinElmer).

### Growth comparison of parent vs bioluminescent SR strains

To characterize the constructed bioluminescent *S*. Reading strains, the growth of each bioluminescent strain was compared to its parent strain. Both parents and bioluminescent *S*. Reading strains were grown overnight in LB agar plates. One colony from each strain was transferred to 5 mL LB broth in 15 mL culture tubes and incubated for 24 h. Both parents and bioluminescent strains were grown in LB alone and LB supplemented with chloramphenicol in a 48-well plate. The overnight cultures were diluted 100-fold, bringing the OD_600_ to approximately 0.13. Four replicates were performed for each strain and blank controls consisting of LB broth only. Optical densities at 600 nm (OD_600_ nm) were measured hourly over a 24-h period, with the temperature maintained at 37°C using BioTek CYTATION one plate reader. The data generated were compared using a Student’s *t*-test with the significant level set at 0.05.

### *In vitro* plasmid stability

Bioluminescent *Salmonella* Reading strains were streaked in LB agar plates with chloramphenicol. Single-colony transfer cultures of each bioluminescent *S*. Reading strain were prepared in LB broth by picking a single colony of *S*. Reading containing the plasmid and inoculating in LB broth without chloramphenicol (four replicates each) and grown overnight at 37°C. Overnight cultures were passed by 1:100 dilution (4 µL in 4 mL LB broth) daily. Cultures starting from days 1 and 2 and continued every other day were serially diluted and spread plated on LB agar plates with and without chloramphenicol until no growth was observed in LB agar plates containing chloramphenicol. Plates were counted after 24 h of incubation. The plasmid stability was determined by calculating the proportion of the colony counts (CFU/mL) from cultures in plates with and without chloramphenicol. The plasmid stability in the presence of chloramphenicol selection was evaluated by passaging bioluminescent *S*. Reading cultures using 1:10 dilution daily. Cultures from days 1 to 7 were serially diluted and plated to determine log reduction in CFU/mL.

### *In vivo* plasmid stability

To determine how long the plasmid could be maintained in both bioluminescent *S*. Reading strains *in vivo*, 144 1 day-old female broiler chicks were obtained from a commercial breeder (IACUC approval number: 22-399). After feather sexing, the chicks were housed in an animal biosafety level 2 facility. The chicks were assigned to three treatment groups with 48 chicks per group. The treatments were *S*. Reading outbreak strain, *S*. Reading non-outbreak strain, and control. After 4 days of acclimatization, chicks from the *Salmonella* treatments were orally administered with 1 mL of 10^8^ CFU/mL respective *S*. Reading strains. Control group chicks received only PBS. Chicks in each treatment group were divided into eight cages, with six chicks per cage. Feed and water were provided to the chicks *ad libitum*. Beginning on the day of the challenge, fecal samples were collected, diluted in PBS, and plated on LB with chloramphenicol to detect the presence of bioluminescent *S*. Reading. Fecal samples were collected over a period of 7 days. Following each fecal sample collection time, the bedding in all treatment cages was replaced with new chick paper to ensure fresh fecal collection. The percentage of fecal positivity for each day was then calculated for each treatment strain.

## RESULTS

### Construction of pBS-*slp*GFP*lux-cat*

**Fig 1:**
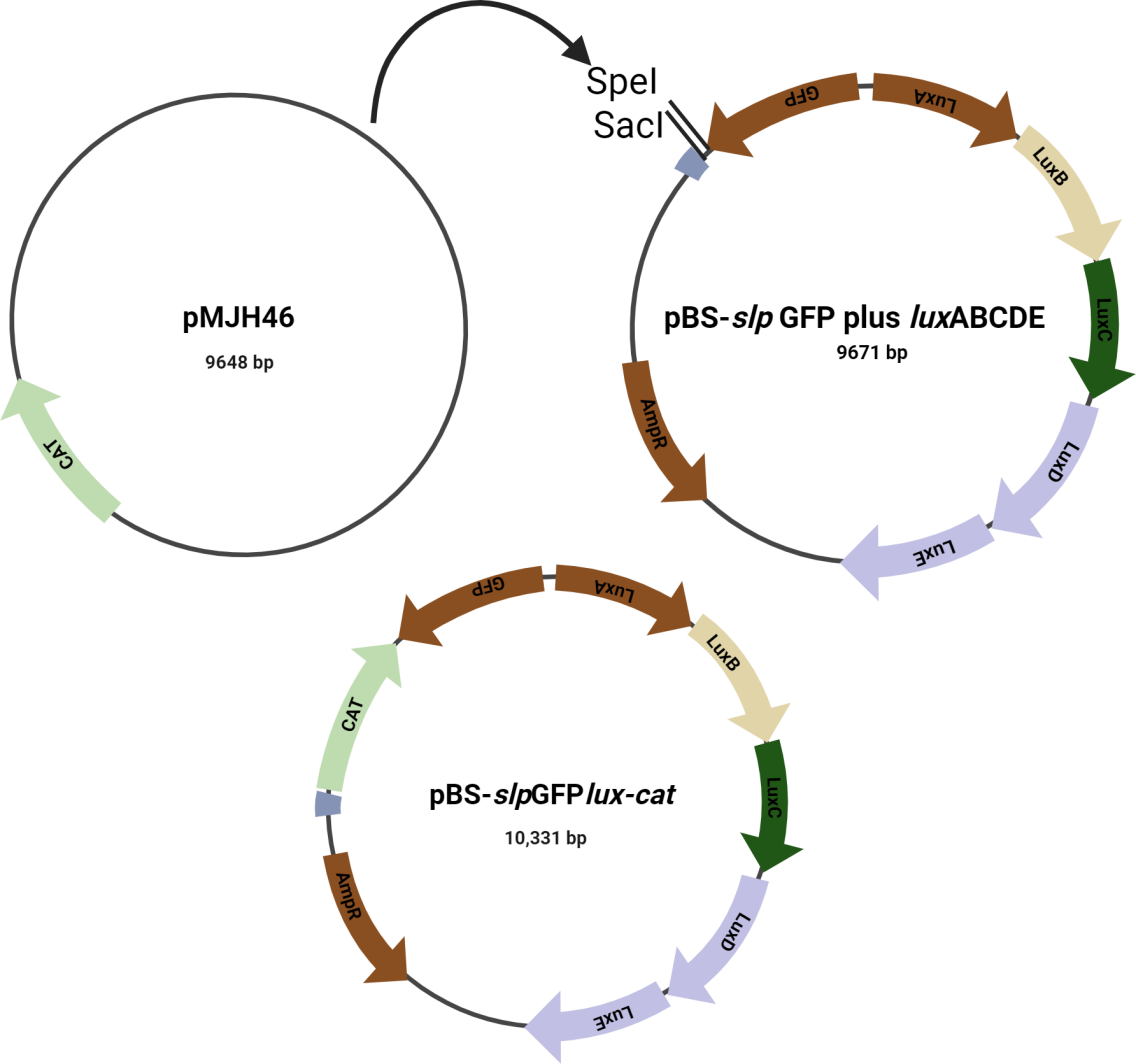
Representation of plasmid manipulation for gene insertion: (**A**) donor plasmid pMJH46 carrying the chloramphenicol resistance gene, (**B**) plasmid pBS-*slp*GFP*luxABCDE* with bioluminescence and ampicillin resistance genes, and (**C**) resulting cloned plasmid pBS-*slp*GFP*lux-cat* incorporating the chloramphenicol resistance gene.

A 717 bp chloramphenicol resistance gene fragment was successfully amplified from pMJH46 (17; Addgene: 67272) and introduced into a plasmid backbone of pBS-*slp*GFP*luxABCDE*. The newly constructed plasmid was named pBS-*slp*GF*Plux-cat*. The size of this plasmid is 10,331 bp and contains chloramphenicol resistance gene, the ampicillin resistance gene, and the bacterial lux operon (*luxCDABE*) genes (Fig. 1).

### S. Reading transformation

**Fig 2:**
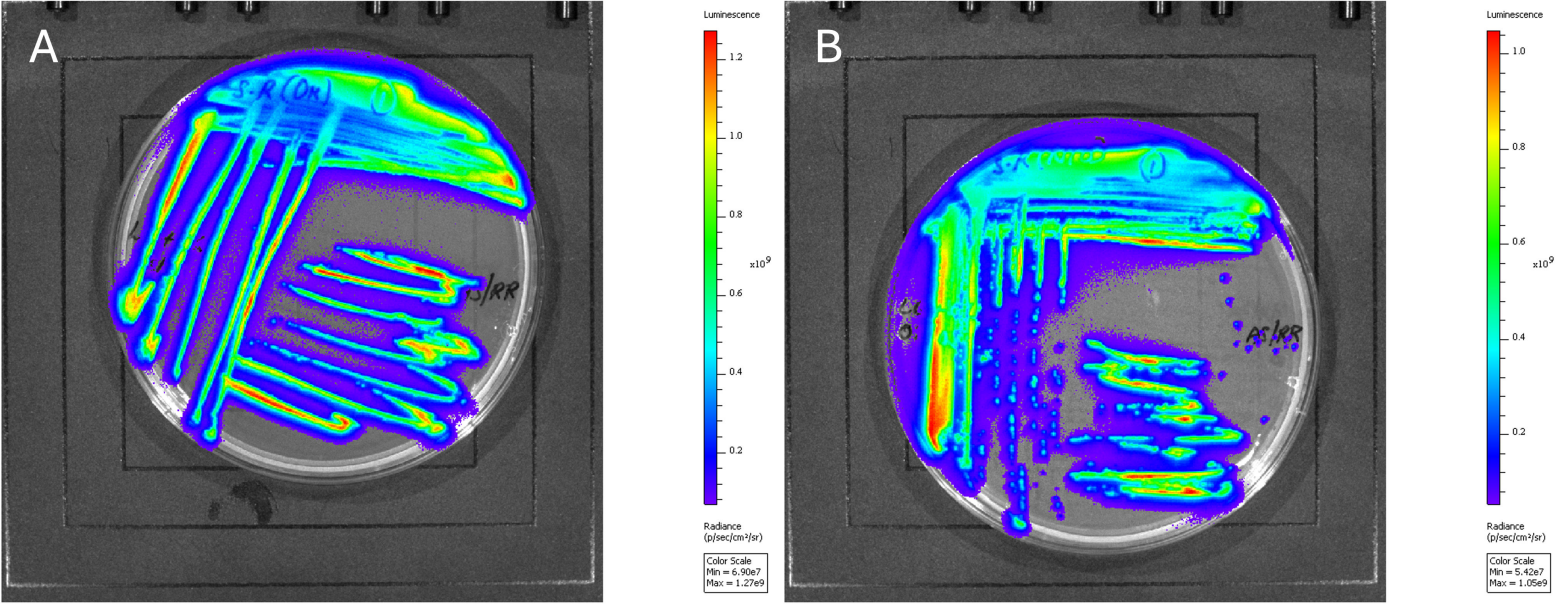
Visualization of bioluminescent *Salmonella* Reading strains: (**A**) outbreak strain and (**B**) non-outbreak strain grown on LB with chloramphenicol. Both strains expressing high levels of bioluminescence of 10^8–9^ photons/s/cm^2^/steradian quantified by *an in vivo* imaging system.

The electrocompetent *S*. Reading strains were successfully transformed with the pBS-*slp*GFP*lux-cat* by electroporation. After 24 h of incubation, bioluminescent colonies were detected using the IVIS, which confirmed the transformation. Quantified by the IVIS, both strains exhibited high bioluminescence levels ranging from 10^8^ to 10^9^ photons/s/cm^2^/sr (Fig. 2).

### Growth comparison of the parent vs bioluminescent *S.* Reading strains

**Fig 3:**
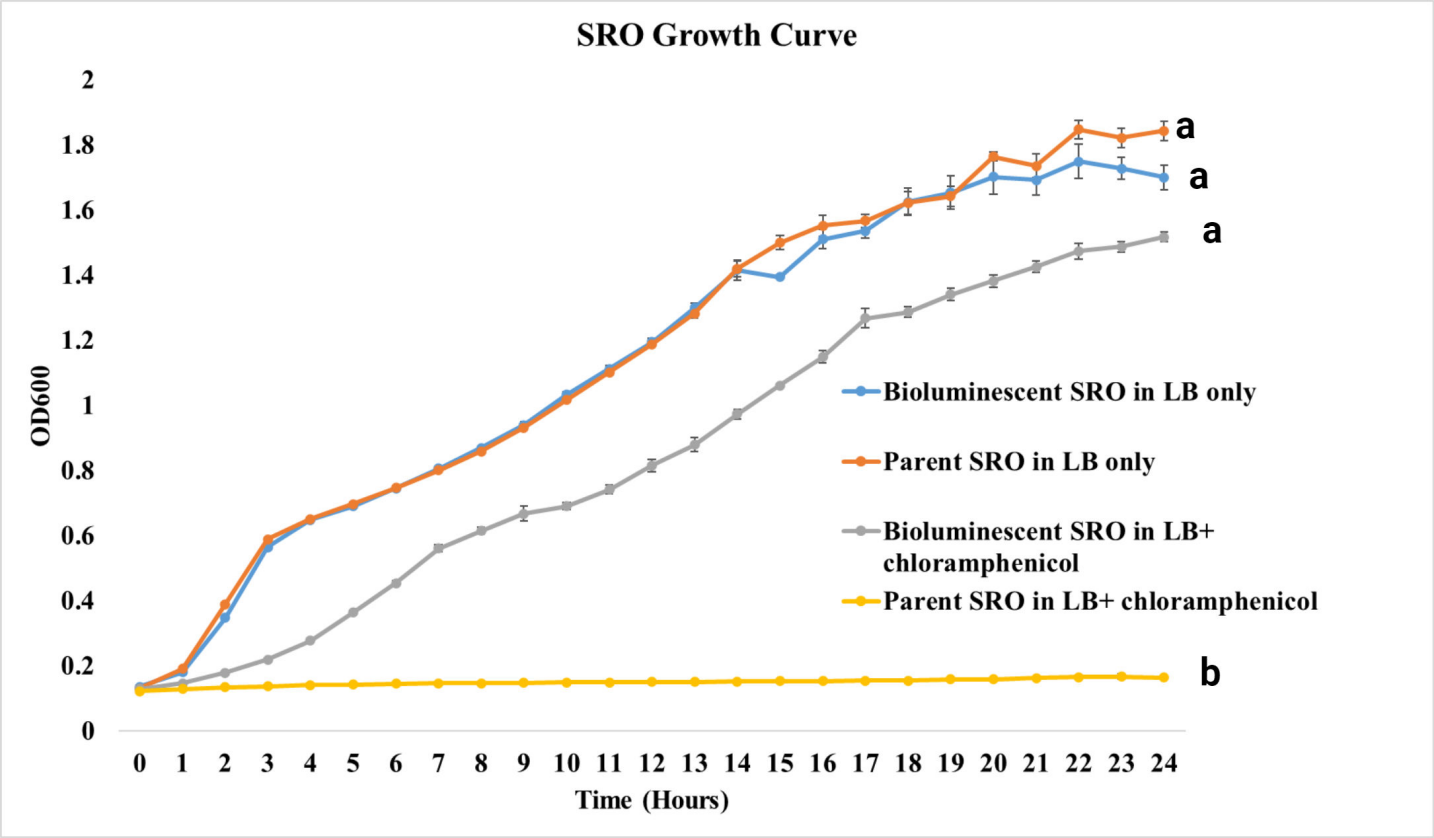
Comparative growth curves of the parent and bioluminescent *Salmonella* Reading outbreak (SRO) strains in LB media with and without addition of chloramphenicol. Differences between parent and bioluminescent strains in both media types were analyzed using Student’s *t*-test (significance level: *P* = 0.05).

**Fig 4:**
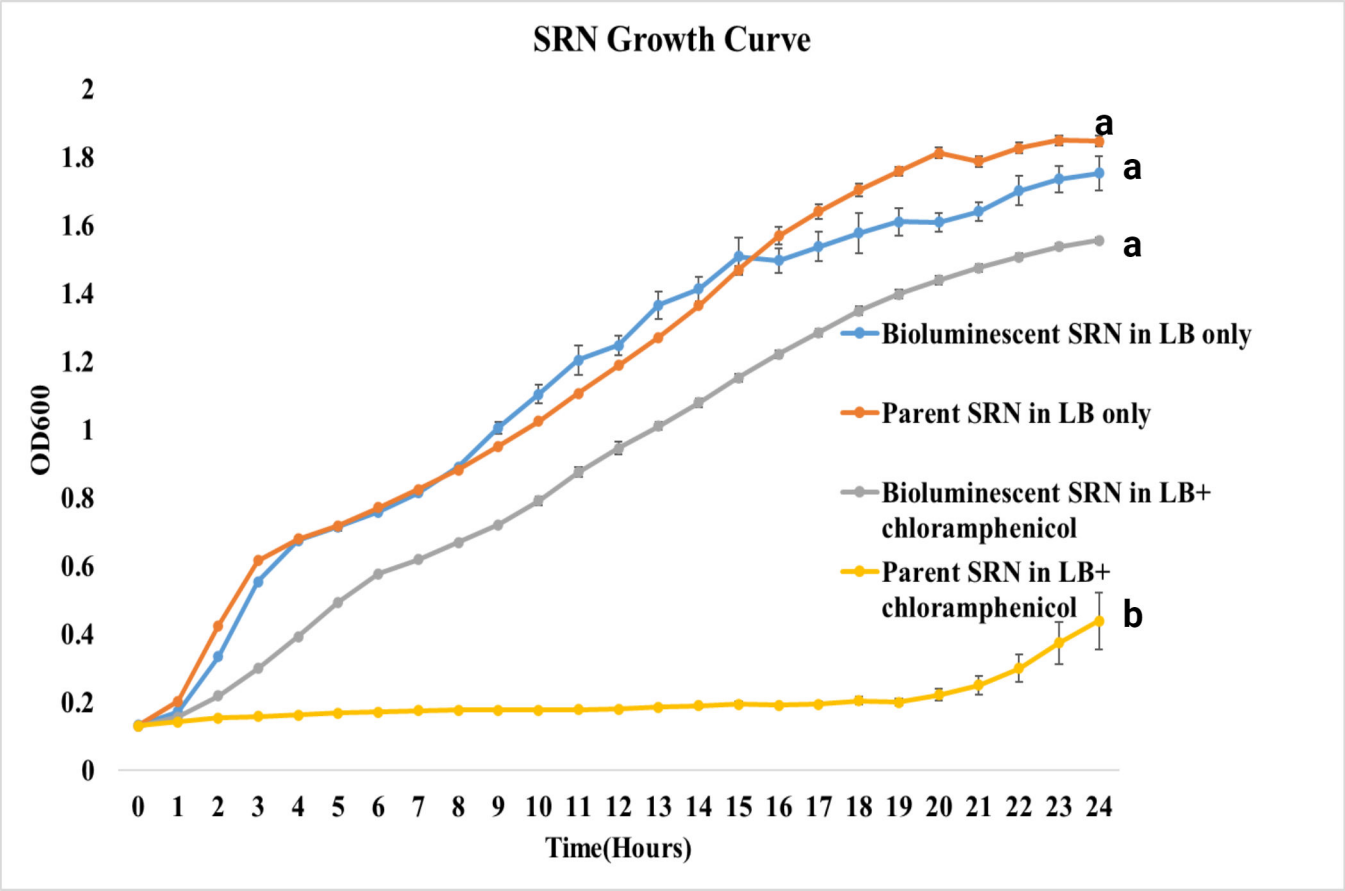
Growth curve comparison of the parent and bioluminescent *Salmonella* Reading non-outbreak (SRN) strains in LB media with and without chloramphenicol. Growth differences between parent and bioluminescent strains under both conditions were performed using Student’s *t*-test (significance level: *P* = 0.05).

Over a 24-h period in LB broth without chloramphenicol, the growth of both parent and bioluminescent SRO strains showed no difference (*P* = 0.87). Similarly, under the same conditions, the parent and bioluminescent SRN demonstrated no difference in growth (*P* = 0.82). However, as expected, both parent strains failed to grow in the presence of chloramphenicol, unlike their bioluminescent counterparts (*P* = <0.001) (Fig. 3 and 4).

### *In vitro* stability of pBS-*slp*GFP*lux-cat*

**Fig 5:**
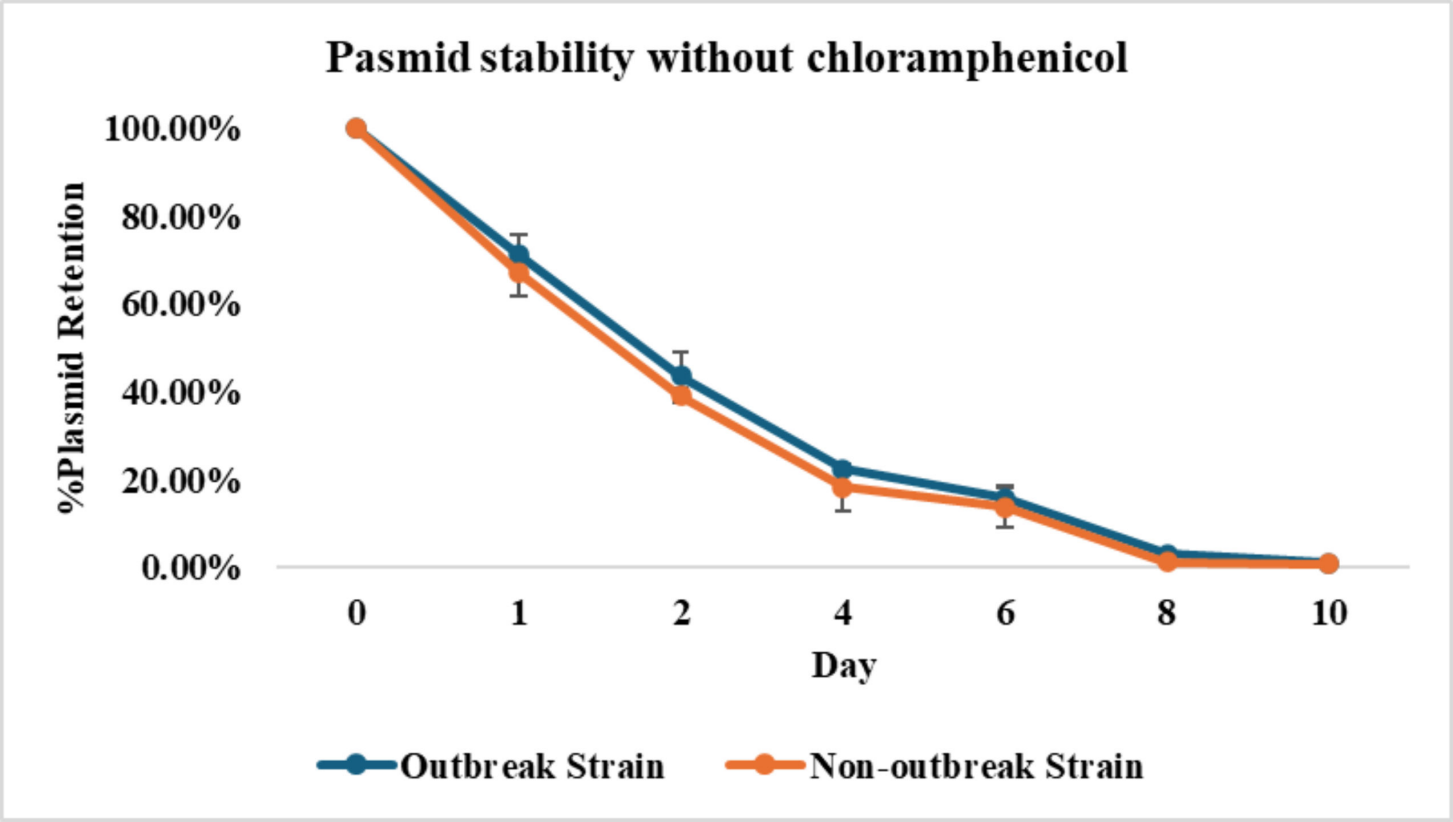
*In vitro* plasmid stability of the constructed bioluminescent *Salmonella* Reading strains without chloramphenicol. Both strain cultures were sub-cultured daily in LB media alone. The figure shows the percent proportion of *Salmonella* Reading that retained the plasmid at each day of passage.

**Fig 6:**
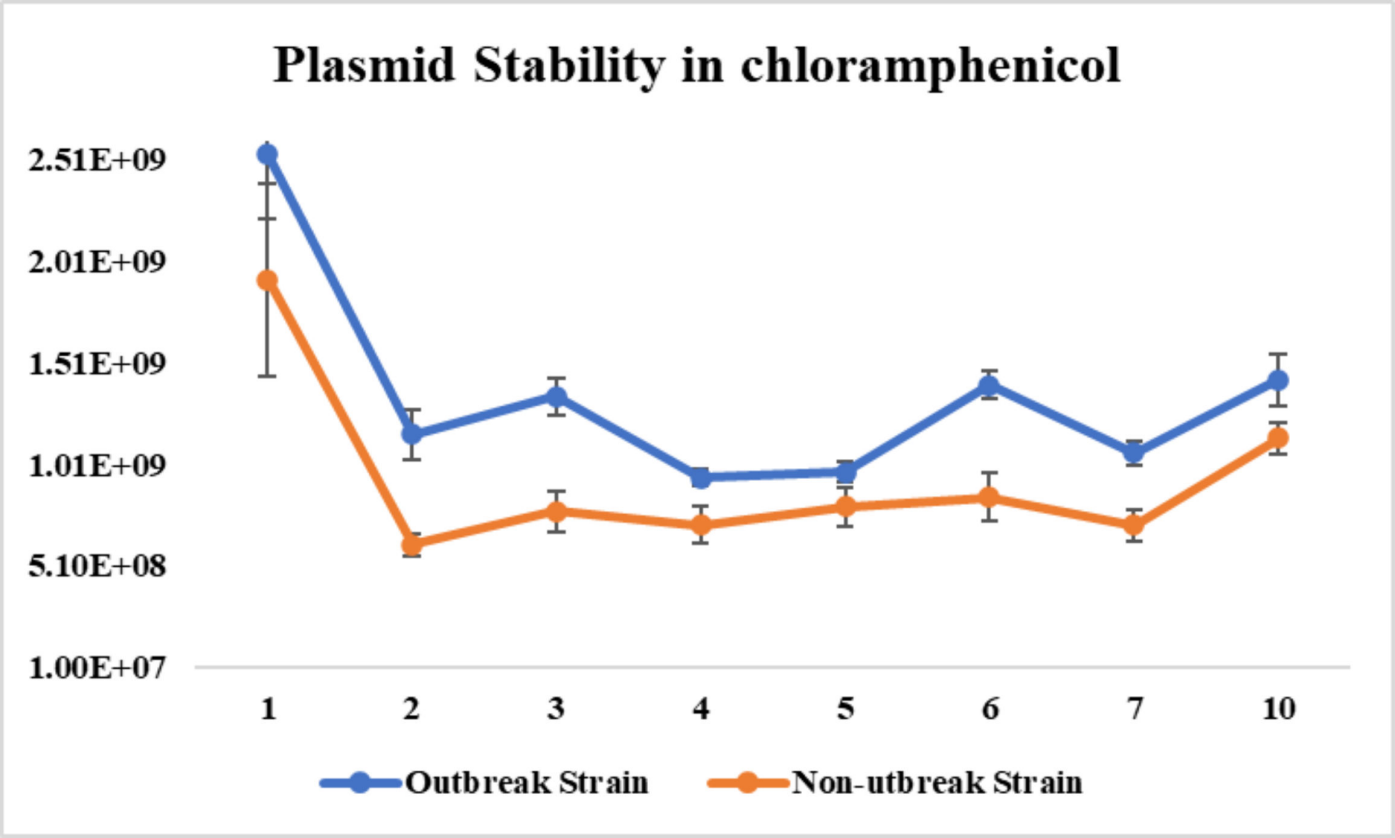
*In vitro* plasmid stability in constructed bioluminescent *Salmonella* Reading (SR) strain cultures daily sub-cultured in LB media supplemented with 10 µg/mL chloramphenicol. The figure shows the reduction in CFU/mL determined in day 1–10 cultures through plating.

The bioluminescent *S*. Reading strains gradually lost the plasmid when grown in the absence of chloramphenicol. By the 10th day of the 100-fold passage, about 1% of the outbreak strain and 0.8% of the non-outbreak strain still maintained the plasmid. However, when grown with chloramphenicol, the strains maintained relatively stable level bacterial counts (CFU/mL) for more than 10 days of testing (Fig. 5 and 6).

### *In vivo* stability of pBS-*slp*GFP*lux-cat* in broiler chicks

**Fig 7:**
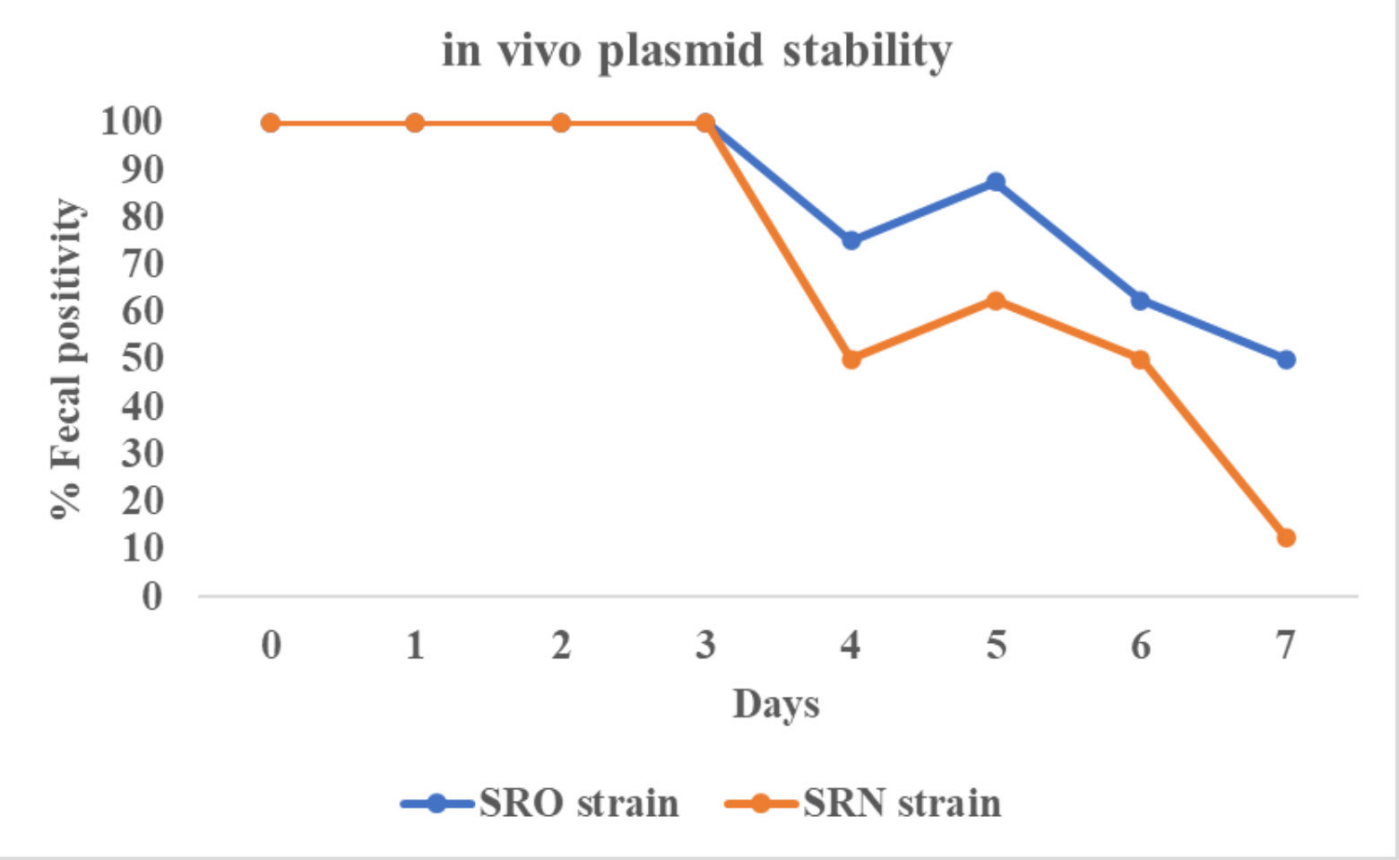
*In vivo* plasmid stability of the constructed bioluminescent *Salmonella* Reading strains in chicks. The graph displays the percent of fecal positivity for bioluminescent *Salmonella* Reading from eight cages per strain with six chicks per cage.

Bioluminescent *S*. Reading strains were traceable in feces of chicks for up to a week following oral challenge. The fecal positivity was at its peak during the first 3 days post-challenge. This was followed by a gradual decline in fecal positivity. By day 7 post-challenge, fecal positivity was approximately 50% in chicks challenged with the SRO strain and about 10% in those with the SRN strain (Fig. 7).

## DISCUSSION

In this study, two *S*. Reading strains were transformed with a bioluminescence marker gene, enabling their use in food safety monitoring employing bioluminescent imaging. This approach can aid in understanding the survival and transmission mechanisms of this newly emerged food-borne pathogen in poultry and poultry products. One of these strains is a reference non-outbreak *S*. Reading strain isolated many years before the recent *S*. Reading outbreak. This strain was not associated with any human illness. The other is the outbreak *S*. Reading strain, which was isolated from the recent *S*. Reading outbreak (7). Whole genome analysis and multilocus sequence typing show that the two strains are genomically distinct, with the outbreak strain having acquired numerous virulence and antimicrobial resistance genes over time (7). As such, making both strains bioluminescent will help in studying their difference in the ability to contaminate products, colonize tissues and spread in poultry.

The bioluminescence marker gene enables bacteria to emit light, which is detectable through bioluminescent imaging. The bacterial lux gene (lux operon) utilized in this study originates from a naturally occurring bacteria, called *Photorhabdus luminescens* (18). The five genes that make up the bacterial lux operon (*luxCDABE*) encode the luciferase enzyme and its aldehyde substrate. Upon expression, luciferase catalyzes an oxidation reaction in bacterial cells, resulting in the emission of visible light (12, 19–22).

Several bacterial species have been tagged with the bacterial lux operon, *luxCDABE*, for various purposes. These include disease monitoring, understanding bacterial colonization and pathogenicity, immunological studies, drug development, and food safety (12, 13, 18, 23–25).

The idea behind making the two *S*. Reading strains bioluminescent is to investigate whether the genes acquired by the outbreak *S*. Reading strain are responsible for its persistence in poultry and wide spread in the North American poultry industry during the recent outbreak. The initial plan was to tag the two *S*. Reading strains with a plasmid pBS-*slp*GFP*luxABCDE* carrying bioluminescence expression and ampicillin resistance genes (13). However, only the non-outbreak strain was successfully tagged with the plasmid. Further investigation revealed that the outbreak strain is resistant to many antibiotics, including ampicillin. As such, it could not be labeled with plasmid pBS-*slp*GFP*luxABCDE*. This affirms the previous finding that the outbreak strain acquired many virulence and antimicrobial resistance genes (7). The newly developed plasmid, pBS-*slp*GFP*lux-cat*, carries bioluminescence, ampicillin resistance, and chloramphenicol resistance genes. It was used to successfully label both *S*. Reading strains. This aligns with a previous study where several *Salmonella* serotypes were successfully tagged with bioluminescent expression plasmids for food safety monitoring purposes (18). However, while most experiments involved commonly isolated *Salmonella* serotypes, to our knowledge, this study is the first to use *S*. Reading. While we did not perform whole-genome sequencing to rule out compensatory mutations following electroporation, the non-integrative nature of the plasmid and its gradual loss over time suggest that chromosomal mutations are unlikely. Future studies incorporating WGS will be valuable in confirming the absence of such mutations.

Both bioluminescent *S*. Reading strains sturdily maintained pBS-*slp*GFP*lux-cat* in the presence of chloramphenicol, supporting the notion of selective antibiotic pressure on plasmids (25). However, in the absence of the selective antibiotic, the percentage plasmid retention declined in both bioluminescent strains, with approximately 1% of both strains still retaining the plasmid after 10 days. Due to the nature of the plasmid stability, the transformed strains would be suitable for short term experimental studies. A different bioluminescence expression plasmid averaged 7 days in various *Salmonella* serotypes (18). Conversely, a similar bioluminescence plasmid exhibits longer stability, lasting 18 days in *Edwardsiella ictaluri*, and much shorter stability of only 72 h in *Aeromonas hydrophilia* (23, 24). This indicates that bacterial genus and species influence plasmid stability.

*In vivo* results demonstrated that the plasmid carrying strains are traceable through fecal shedding in broiler chicks following the oral challenge. Both bioluminescent strains were traceable in feces for up to 7 days using BLI. Although a study shows that *Salmonella* shedding in feces is typically sustained for many days post-oral challenge in poultry (26), the observed decline in fecal shedding of the bioluminescent strains in this study suggests that the bacteria are gradually losing the plasmid. This leads to the plasmid-free *Salmonella* Reading not being able to grow in chloramphenicol selective condition because they are ordinarily not resistant to the antibiotic. Additionally, they do not express bioluminescence and, therefore, cannot be detected by BLI. Future studies should include culturing fecal samples on non-selective media to distinguish between plasmid loss and bacterial clearance. We advise caution in interpreting comparisons between the outbreak and non-outbreak strains due to these limitations. A trend of lower fecal positivity was observed for the SRN strain compared to the SRO strain from days 4 to 7. Given the plasmid instability and the inability to determine whether the decline in fecal positivity was due to plasmid loss or bacterial clearance, no statistical analysis was conducted to compare the two strains. Future studies incorporating methods to distinguish between plasmid loss and bacterial clearance, such as culturing on non-selective media or using chromosomally integrated markers, would allow for more accurate comparisons between strains.

There was impact on growth observed when comparing parent *S*. Reading strains to their bioluminescent counterparts, which aligns with similar findings observed in bioluminescent *E. ictaluri* (14, 15, 23). This confirms that tagging bacteria with a bioluminescent gene does not impose a substantial metabolic burden, thus having no significant effect on their growth. The significant difference seen in growth between the parent and bioluminescent strains in antibiotic-selective conditions is expected. The growth measurement procedure can be reliably used for testing both antimicrobial resistance and the success of bacterial transformation.

### Conclusion

This research shows the efficacy of tagging foodborne pathogens with bioluminescence genes, as well as bioluminescent imaging as a breakthrough method that can be used for easy tracking and monitoring pathogens in poultry and poultry products. The constructed bioluminescent *S*. Reading developed in this study can be used to get invaluable insights into the pathogen’s transmission routes, colonization patterns, and points of meat contamination. These findings could significantly advance our understanding of the pathogen’s behavior and developing effective food safety methods in poultry production to prevent the spread and future outbreaks caused by the pathogen.

## References

[1] MacKenzie KD, Palmer MB, Köster WL, White AP. 2017. Examining the link between biofilm formation and the ability of pathogenic Salmonella strains to colonize multiple host species. Front Vet Sci 4:138. doi:10.3389/fvets.2017.00138 29159172 PMC5581909

[2] Gal-Mor O, Boyle EC, Grassl GA. 2014. Same species, different diseases: how and why typhoidal and non-typhoidal Salmonella enterica serovars differ. Front Microbiol 5:391. doi:10.3389/fmicb.2014.00391 25136336 PMC4120697

[3] Wibisono FM, Wibison FJ, Effendi MH, Plumeriastuti H, Hidayatullah AR, Hartadi EB, Sofiana ED. 2020. A review of Salmonellosis on poultry farms: public health importance. Syst Rev Pharm 11:481–486. doi:10.31838/srp.2020.9.69

[4] O’Bryan CA, Ricke SC, Marcy JA. 2022. Public health impact of Salmonella spp. on raw poultry: current concepts and future prospects in the United States. Food Control 132:108539. doi:10.1016/j.foodcont.2021.108539

[5] Whyte P, Mc Gill K, Collins JD, Gormley E. 2002. The prevalence and PCR detection of Salmonella contamination in raw poultry. Vet Microbiol 89:53–60. doi:10.1016/s0378-1135(02)00160-8 12223162

[6] Shah DH, Paul NC, Sischo WC, Crespo R, Guard J. 2017. Population dynamics and antimicrobial resistance of the most prevalent poultry-associated Salmonella serotypes. Poult Sci 96:687–702. doi:10.3382/ps/pew342 27665007

[7] Miller EA, Elnekave E, Flores-Figueroa C, Johnson A, Kearney A, Munoz-Aguayo J, Tagg KA, Tschetter L, Weber BP, Nadon CA, Boxrud D, Singer RS, Folster JP, Johnson TJ. 2020. Emergence of a novel Salmonella enterica serotype reading clonal group is linked to its expansion in commercial Turkey production, resulting in unanticipated human illness in north America. mSphere 5:10–1128. doi:10.1128/mSphere.00056-20 PMC7160679 32295868

[8] Tanguay F, Vrbova L, Anderson M, Whitfield Y, Macdonald L, Tschetter L, Hexemer A. 2017. Outbreak of Salmonella reading in persons of eastern mediterranean origin in Canada, 2014–2015. Can Commun Dis Rep 43:14–20. doi:10.14745/ccdr.v43i01a03 29770042 PMC5757717

[9] Public Health Agency of Canada. 2020. Public health notice — Outbreak of Salmonella illnesses linked to raw turkey and raw chicken. Available from: https://www.canada.ca/en/public-health/services/public-health-notices/2018/outbreak-Salmonella-illnesses-raw-turkey-raw-chicken.html

[10] CDC. 2019. Outbreak of multidrug-resistant Salmonella infections linked to raw Turkey products. Available from: https://www.cdc.gov/salmonella/reading-07-18/index.html

[11] Contag CH, Contag PR, Mullins JI, Spilman SD, Stevenson DK, Benaron DA. 1995. Photonic detection of bacterial pathogens in living hosts. Mol Microbiol 18:593–603. doi:10.1111/j.1365-2958.1995.mmi_18040593.x 8817482

[12] Troy T, Jekic-McMullen D, Sambucetti L, Rice B. 2004. Quantitative comparison of the sensitivity of detection of fluorescent and bioluminescent reporters in animal models. Mol Imaging 3:9–23. doi:10.1162/15353500200403196 15142408

[13] Castañeda CD, McDaniel CD, Abdelhamed H, Karsi A, Kiess AS. 2019. Evaluating bacterial colonization of a developing broiler embryo after in ovo injection with a bioluminescent bacteria. Poult Sci 98:2997–3006. doi:10.3382/ps/pez053 30789222

[14] Beyer W, Böhm R. 1996. Labeling Salmonella live vaccine strains with the lux operon from Vibrio fischeri improves their detection and discrimination from wild type. Microbiol Res 151:407–419. doi:10.1016/S0944-5013(96)80011-5 9022302

[15] Bautista DA, Chen J, Barbut S, Griffiths MW. 1998. Use of an autobioluminescent Salmonella hadar to monitor the effects of acid and temperature treatments on cell survival and viability on lactic acid-treated poultry carcasses. J Food Prot 61:1439–1445. doi:10.4315/0362-028x-61.11.1439 9829182

[16] Thames HT, Pokhrel D, Willis E, Rivers O, Dinh TTN, Zhang L, Schilling MW, Ramachandran R, White S, Sukumaran AT. 2023. Salmonella biofilm formation under fluidic shear stress on different surface materials. Foods 12:1918. doi:10.3390/foods12091918 37174455 PMC10178852

[17] Hossain MJ, Thurlow CM, Sun D, Nasrin S, Liles MR. 2015. Genome modifications and cloning using a conjugally transferable recombineering system. Biotechnol Rep (Amst) 8:24–35. doi:10.1016/j.btre.2015.08.005 28352570 PMC4980705

[18] Karsi A, Howe K, Kirkpatrick TB, Wills R, Bailey RH, Lawrence ML. 2008. Development of bioluminescent Salmonella strains for use in food safety. BMC Microbiol 8:1–9. doi:10.1186/1471-2180-8-10 18211715 PMC2257966

[19] Frackman SUSAN, Anhalt MICHAEL, Nealson KH. 1990. Cloning, organization, and expression of the bioluminescence genes of Xenorhabdus luminescens. J Bacteriol 172:5767–5773. doi:10.1128/jb.172.10.5767-5773.1990 2211511 PMC526893

[20] Meighen EA. 1993. Bacterial bioluminescence: organization, regulation, and application of the lux genes. FASEB J 7:1016–1022. doi:10.1096/fasebj.7.11.8370470 8370470

[21] Hastings JW. 1996. Chemistries and colors of bioluminescent reactions: a review. Gene 173:5–11. doi:10.1016/0378-1119(95)00676-1 8707056

[22] Wilson T, Hastings JW. 1998. Bioluminescence. Annu Rev Cell Dev Biol 14:197–230. doi:10.1146/annurev.cellbio.14.1.197 9891783

[23] Ozdemir E, Abdelhamed H, Ozdemir O, Lawrence M, Karsi A. 2023. Development of bioluminescent virulent Aeromonas hydrophila for understanding pathogenicity. Pathogens 12:670. doi:10.3390/pathogens12050670 37242340 PMC10222170

[24] Karsi A, Menanteau-Ledouble S, Lawrence ML. 2006. Development of bioluminescent Edwardsiella ictaluri for noninvasive disease monitoring. FEMS Microbiol Lett 260:216–223. doi:10.1111/j.1574-6968.2006.00310.x 16842347

[25] Subbiah M, Top EM, Shah DH, Call DR. 2011. Selection pressure required for long-term persistence of bla_CMY-2_-positive IncA/C plasmids. Appl Environ Microbiol 77:4486–4493. doi:10.1128/AEM.02788-10 21602382 PMC3127679

[26] Van Immerseel F, De Buck J, Pasmans F, Bohez L, Boyen F, Haesebrouck F, Ducatelle R. 2004. Intermittent long-term shedding and induction of carrier birds after infection of chickens early posthatch with a low or high dose of Salmonella enteritidis. Poult Sci 83:1911–1916. doi:10.1093/ps/83.11.1911 15554070

